# Microstructure and Mechanical Properties of Pressure-Quenched SS304 Stainless Steel

**DOI:** 10.3390/ma12020290

**Published:** 2019-01-17

**Authors:** Peng Wang, Yang Zhang, Dongli Yu

**Affiliations:** 1 State Key Laboratory of Metastable Materials Science and Technology, Yanshan University, Qinhuangdao 066004, China; wp@ysu.edu.cn; 2 Key Laboratory for Microstructural Material Physics of Hebei Province, School of Science, Yanshan University, Qinhuangdao 066004, China; zyang9393@gmail.com

**Keywords:** SS304 stainless steel, pressure quenching, ultrafine microstructure, mechanical properties

## Abstract

Bulk SS304 polycrystalline materials with ultrafine microstructures were prepared via a high-pressure self-heating melting and quenching method. Analyses of phase composition, grain size and microstructure were performed using metallographic analysis, X-ray diffraction, Rietveld refinement and transmission electron microscope (TEM). The effects of pressure and cooling rate on the solidification of SS304 were analyzed. Mechanical property test results show that, compared with the as-received sample, the hardness and the yield strength of the pressure-quenched (PQ) samples were greatly increased, the ultimate tensile strength changed minimally, and the elongation rate became small, primarily due to the large density of dislocations in the sample. The high-pressure self-heating melting and quenching method is an exotic route to process a small piece of steel with moderate properties and ultrafine microstructure.

## 1. Introduction

SS304 stainless steel is one of the most widely used Ni/Cr-based stainless steels and offers good processing performance, high ductility and corrosion resistance [1,2,3,4,5], but the low hardness and strength of this material limit its application in the constructional field. Grain refininghasbeen confirmed as being able toimprove the strength and ductility of stainless steel [6,7,8,9].

Bulk ultrafine-grained materials have beenprepared by mechanical alloying and sintering [10,11,12]. In the process of mechanical alloying, the powder is easily oxidized, which affects the performance of the final sample. Together with the use of high-purity inert gas evaporation and condensation in preparation of the nanocrystalline precursor, the in situ compaction method can produce clean nanocrystalline materials [13]. However, the sample prepared with this method has high porosity and low density, and it is difficult to prepare large samples.

Pressure quenching (PQ) of a liquid alloy is a good method for preparation of bulk nanomaterials [14,15,16,17,18]. The material is first heated to melting under pressure, and quenching solidification is performed from the molten state. The resulting sample exhibits notably clean grain boundaries and no internal porous voids. Because the pressure shortens the inter-atomic distance adjacent to the electron orbital overlap, the crystal structure of the materials, the electronic structure and the interaction between atoms (molecule) are changed, and these changes affect certain material properties (such as melting temperature, thermal conductivity, density, etc.) [19,20]. During solidification, pressure promotes the formation of crystal nuclei in the molten alloy and restrains atomic diffusion such that limited crystal nuclei growth can occur, which is advantageous for preparation of ultrafine-grained bulk materials.

In this work, a SS304 stainless steel column and a high-pressure self-heating melting and quenching method were used to prepare the bulk ultra fine-grained stainless steel, which was characterized via X-ray diffraction (XRD), scanning electron microscope (SEM) and transmission electron microscope (TEM). The effects of pressure and cooling rate on the solidification of SS304 were analyzed. The hardness and tensile test results show that the hardness and yield strength of the PQ samples weregreatly increased relative to conventional solid solution water quenching samples.

## 2. Materials and Methods

### 2.1. Experimental Materials

The starting material used in this study consisted of SS304 stainless steel rods with a diameter of 25 mm. The chemical composition listed in Table 1 was determined by X-ray fluorescence method and infra-redcarbon–sulfur spectrometer.

### 2.2. Experimental Procedures

As-received SS304 rod was packaged in an evacuated quartz glass capsule then heat treated at 1500 °C for 15 min in furnace and water quenched to prepare as-cast SS304.

**Figure 1:**
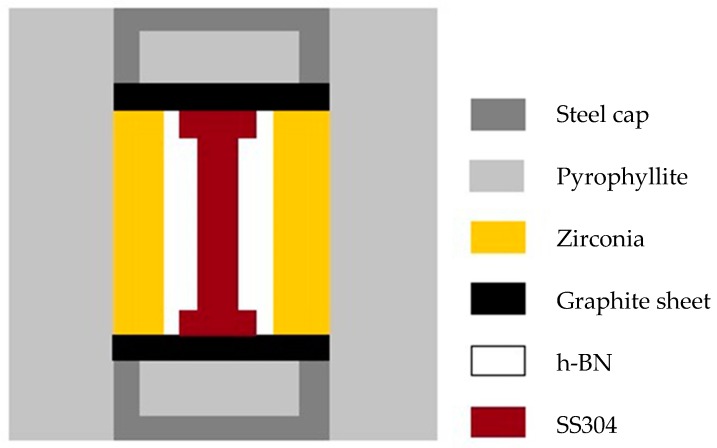
Sketch map of the PQ sample assembly.

Figure 1 shows the sketch map of the PQ sample assembly. The starting materials were cut from the rod specimen with a diameter of 5.5 mm and a length of 10.5 mm via electrical discharge wire cutting. A mini-lathe was used to cut off the specimen’s oxide skin. The final prepared samples with a diameter of 5 mm and a length of 10 mm were fine columnar samples with a bright surface.

The samples were placed in a hexagonal boron nitride (h-BN) crucible (outer diameter of 10 mm), and set out on zirconia thermal insulation sleeve with a wall thickness of 2 mm. PQ experiments were performed using a China-type large volume cubic press with a maximum of 800 tons for every WC anvil [21]. The samples were first compressed to the required pressure and subsequently heated to the desired temperature for 10 min via resistive heating. For all experiments, the temperature was measured in situ with a type C thermocouple (W5/Re26). The pressure was estimated using previously obtained calibration curves based on the change in electrical resistance [22] during the phase transitions of Bi at room temperature. After heating, the samples were quenched to room temperature at a cooling rate of ~200 K/s before the pressure was removed. Samples recovered under ambient conditions showed a bulgy middle section with obvious signs of melting.

Vickers hardness testing was performed on a micro-hardness tester (FUTURE-TECH, FM-ARS9000) (Kanagawa Prefecture, Japan) with a load of 200 gf for 10 s. The hardness of each sample was tested on at least 12 indentations, and the average values and the standard deviation were calculated. 

The as-received and PQ SS304 stainless steel samples were cut to rod tensile specimens with a gauge length of 4 mm and a cross-sectional diameter of 2 mm. Limited by our existing preparation conditions, the PQ sample has only 3 mm cross-sectional diameter, which makes it impossible to carry out tensile tests. We did a tensile test along the axis. The tensile tests were performed on the samples at a nominal strain rate of 5 × 10^−4^ at room temperature in air. The tests were performed with a servo-hydraulic tensile testing machine (Hegewald & Peschke, Inspekt table 100) (Nossen, Germany), and an optical extensometer (Beijing, China) was used to measure the elongation.

Phase identification analysis was performed using X-ray diffraction (XRD, Rigaku, D/max-2500/PC) (Tokyo, Japan) with CuKa radiation. Prior to XRD examination, the surfaces of the samples were ground with SiC emery papers to a roughness of 2000 and electro-polished with an electrolyte containing 90% acetic acid and 10% perchloric acid at 17 V for 300 s. All of the XRD profiles were normalized according to the max value. The Maud software for performing Rietveld refinement was used to calculate the grain size and phase fractions [23] Confidence factor of those fitting were: Rw < 15.0, sig < 2.0. Thus, this accuracy coincided with grain size test standard.

Scanning electron microscopy (SEM, HITACHI S-4800) (Tokyo, Japan) with an energy dispersive spectrometer (EDS) was used to observe the microstructures and chemical compositions of the samples.

The microstructures of the PQ specimens were observed using transmission electron microscopy (TEM, JEOL 2010) (Tokyo, Japan). The specimens were first mechanically thinned to 50 μm and subsequently electro-polished with twin jet equipment (Struers, TenuPol-5) (Ballerup, Denmark) at 17 V androom temperature. The electrolyte used contained 90% acetic acid and 10% perchloric acid.

## 3. Results and Discussion

### 3.1. Microstructure

**Figure 2:**
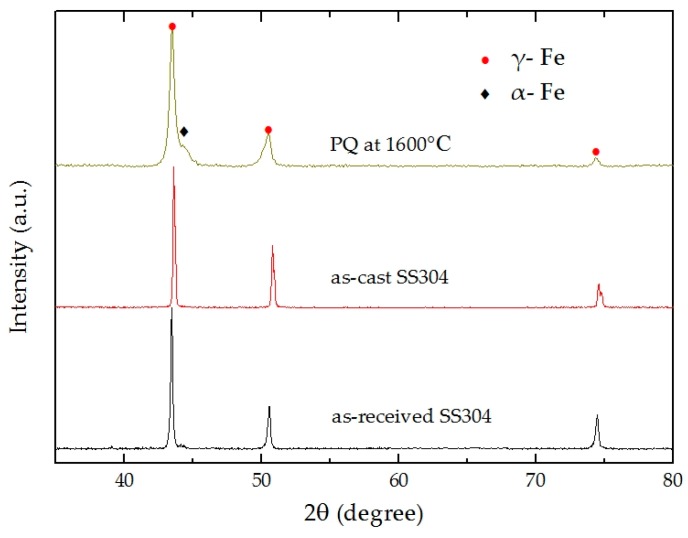
X-ray diffraction (XRD) spectra of the as-received SS304, as-cast SS304 and PQ SS304.

Figure 2 shows X-ray profiles for the as-received SS304, as-cast SS304 and PQ samples of SS304 at 4 GPa and 1600 °C. The microstructure of the high-pressure melting specimen containsγ-Fe [face-centered cubic (FCC)] and α- Fe [body-centered cubic (BCC)].

Figure 2 shows that the diffraction peaks of the PQ sample are obviously wider than those of the other samples, which might be due to grain refinement and microstrain caused by the solidification process. Rietveld refinements were performed using the MAUD software on the high-pressure melting specimen’s XRD data. From the results, the weight percent of the γ-Fe phase is approximately 92.7%, the grain size is 567 nm, and the microstrain is 5.3 × 10^−3^, and the weight percent of the α- Fe phase is near 7.3%.

**Figure 3:**
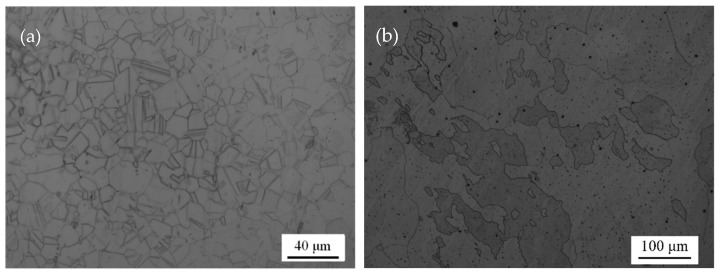
Metallographic image: (**a**) as-received SS304; and (**b**) as-cast SS304.

Figure 3a shows the metallographic results of the as-received SS304. This material displays a typical single-phase austenitic water quenched structure, with a grain size of approximately 30 μm and uniform equiaxed grains. 

Figure 3b shows the metallographic results of the as-cast SS304. Combined with the XRD result, we know that the as-cast SS304 has a single-phase austenitic structure, with a coarse grain size and irregular grain shape.

**Figure 4:**
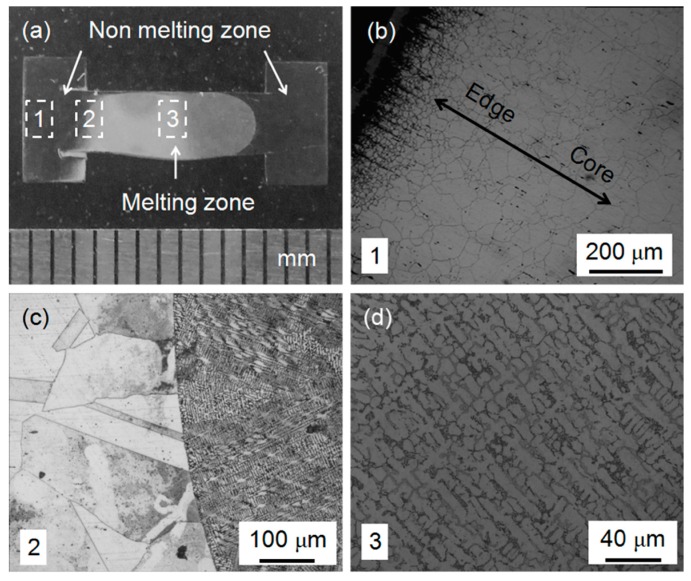
Photo and metallographic data of PQ SS304: (**a**) photo of PQ SS304; and (**b**–**d**) metallographic data of Selected Areas 1–3 of PQ SS304, respectively.

A photo and metallographic data for PQ SS304 are shown in Figure 4. The PQ SS304 sample was split from the middle and ground, polished and etched. Figure 4a shows a section of this material. The sample is clearly split into three pieces by two arcs. The melting zone is located in the middle with a non-melting zone around the outside. Metallographic images of the three areas are shown in Figure 4b–d.

Figure 4b is located on the edge of the sample. In the experiment, the sample was spontaneously heated under the action of an electric current, and, because the cross-sectional area of both ends is larger, the current density was small, the temperature was low, and no melting occurred. The current density of the core is large, which generates high temperature. Taking into account the role of heat conduction, the temperature of Selected Area 1 is gradually increased from the left to the right.

The non-melting zone experienced a process known as high-pressure solid solution quenching. Grain growth occurred in this process and, the closer to the core, the higher the temperature, and the larger the final grain size produced. This behavior is consistent with the result, which shows that the grain size is gradually increased from the edge to the core area in Figure 4b.

Figure 4c illustrates a clear arc boundary, in which the left side shows the larger grain with a shape similar to that of the as-received SS304 but larger in size. On the right side of the dividing line is an ultrafine structure obtained after melting and quenching under high pressure. There are many large grains with dendritic structure inside. The dendritic structure within each grain was observed to have obvious orientation. However, in the whole melting region, there are many grains with different angle distribution, which leads to the weakening of the overall orientation of the sample. Comparing the intensity ratio of the diffraction peaks in the XRD pattern in Figure 2, we can see that small degree of texture exists. Analyses of the texture with metallographic image and XRD showed good agreement.

Combined with the XRD analysis, the samples were characterized as nearly single-phase γ-Fe containing only a small amount of α- Fe. Thus, it can be concluded that the microstructure of the two forms in Figure 4b is austenitic, although the shapes are not the same.

Figure 4d displays a photo of the metallographic structure of the melted zone. The γ-Fe grains show dendritic distribution. We measured the primary and secondary dendritic arm spacing of about 200 dendrite arms in the core and edge regions of melting zone. The average size of primary dendritic arm spacing is 4.54 microns and secondary dendritic arm spacing is 8.67 microns [24].

**Figure 5:**
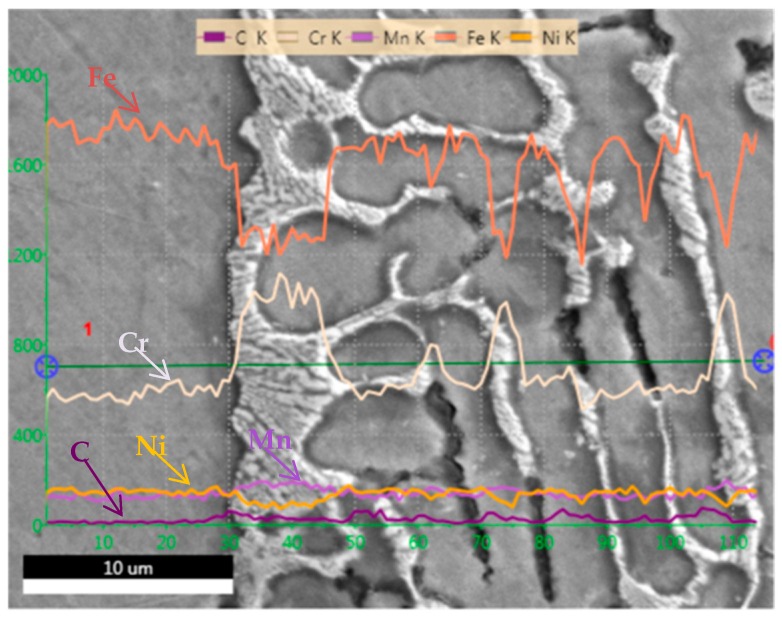
SEM-EDS analysis of PQ SS304.

As shown in Figure 5, we performed SEM-EDS analysis of PQ samples. The chemical composition of non-melting region, dendrite and interdendritic regions were analyzed by line scanning. The results show that the chemical composition of dendrite is basically the same as that of non-melting region. However, in the interdendritic region, the contents of Fe and Ni decreased, while the contents of Cr and Mn increased. This is due to segregation during solidification. From the SEM morphology analysis, the segregation of alloying elements also results in a significant difference in interdendritic contrast with dendrites and non-melting regions. Combining with the XRD results in Figure 2, it can be determined that the α phase exists in the interdendritic region.

Compared with Figure 3, Figure 4 and Figure 5, the metallographic structure is greatly different, which is due to the tremendous pressure and the cooling rate that affect the solidification of SS304. Due to the pressure, the nucleation rate of the melt was increased, and the diffusion of atoms required for grain growth was suppressed, which wasfavorable for formation of a fine-grained structure. In this experiment, the spontaneous heating method for the sample created a high heating efficiency, which limited the heat in the sample itself, and the total calorific value was small. After the heating wasstopped, the temperature of the sample decreased sharply at a rate of approximately 200 K/s. In the process of rapid cooling, a large degree of super-cooling occurred, which resulted in the formation of fine dendrites. In the end, a small dendritic crystal was formed under the combined effect of pressure and cooling rate.

**Figure 6:**
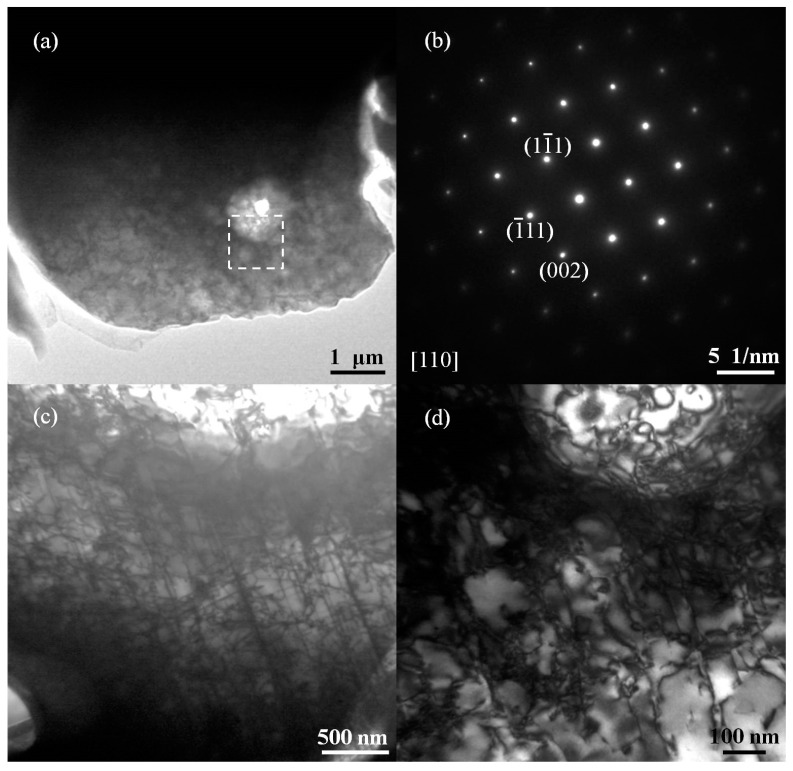
TEM image of the melting zone in the PQ sample: (**a**) bright field image of the melting zone in the PQ sample; (**b**) selected area electron diffraction; (**c**) bright-field image of the selected area; and (**d**) dark-field image of the selected area.

Figure 6 shows a TEM image of the melting zone in the PQ sample. The bright-field image of Figure 6a shows that the observed region is a single crystal, which can be confirmed by the selected area electron diffraction spots shown in Figure 6b. According to the index of calibration, the single crystal is a face-centered cubic structure and is a γ-Fe grain. It can be observed in Figure 6a that many microstructures are present in the single crystal. Figure 6c,d shows an enlarged selected area of the bright-field and dark-field image. We note the presence of many dislocations in the single crystal due to the high pressure, under which the atomic spacing becamesmaller and created crystal lattice distortion. In the quenching process, due to the rapid decline in temperature, the atom diffused slowly, which formedmany dislocation defects. The dislocation entanglements together formed the sub-grain boundary, and the single crystal was divided into many nano-scale sub-grains. The average sub-grain size of this melted area is approximately 100 nm. It is far less than the average grain size calculated using Rietveld refinement of the whole sample.

### 3.2. Mechanical Properties

The melting and non-melting zones of the PQ samples and the as-received samples were tested to determine their Vickers microhardness, and the results are listed in Table 2. The average hardness of the melted zone of the PQ sample wasincreased by 33% compared with that of the as-received sample. This result is primarily due to the high density of the samples prepared by high pressure and grain refinement. The hardness of the non-melted zone of the PQ sample wasslightly decreased, which might be caused by the growth of the grain size.

Table 3 shows the tensile test results for the as-received sample and the PQ sample. The yield strength of the PQ samples was increased by 86.7% compared with the as-received sample, which is consistent with the results of the hardness test. In Figure 6, we note that the dislocation density of the PQ samples is quite large. Under tension, the dislocations become entangled with each other, a process that affects the slip, and thus the yield strength increases. However, the high density of the samples prepared by high pressure and grain refinement canalso lead to an increase in the yield strength. The ultimate tensile strength is the maximum stress that a material can withstand, and this value displayed minimal change in this experiment. The large number of dislocations makes deformation of the PQ samples difficult, and the elongation rate decreases.

**Figure 7:**
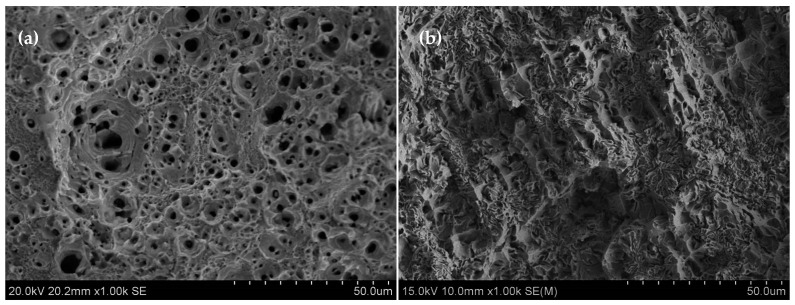
SEM image of tensile fracture: (**a**) as received SS304; and (**b**) pressure quenching SS304.

Figure 7 shows an SEM image of the tensile fracture. In Figure 7a, no split source is observed on the surface of the tensile fracture plane of the as-received sample. Many deep circular equi-axed dimples exist, which shows that the sample does not contain any obvious stress concentration, and the ductility is good. This result is consistent with the tensile test results in Table 3. The dimple sizes are not uniform; some are quite large, others are rather small, and the difference is great. However, the distribution of large and small dimples on the fracture surface is quite uniform and presents the obvious characteristics of aggregation and growth. The fracture type is micro-aggregation-type ductile fracture.

Many shallow and long strip dimples are visible in Figure 7b, and no significant second-phase particles are found in the majority of small dimples. The dimple size and arrangement are similar to the grain size and arrangement in Figure 4d. It is known that many dislocation defects are contained in this sample. Under the action of tensile stress, the dislocations aggregate to the grain boundaries, and stress concentration is formed. Intergranular fracture occurred before the dimples grew. The PQ sample shows the combined characteristics of brittle and ductile properties, and the ductility is poor.

## 4. Conclusions

(1) Bulk SS304 polycrystalline material with an ultrafine microstructure was prepared using the PQ method at 4 GPa and 1600 °C. TEM analysis showedthat the dislocation density in the melting zone was quite large, and the sub-grain size formed by these dislocations was approximately 100 nm.

(2) Mechanical property test results show that, compared with the as-received sample, the hardness of the PQ samples increased by 33%, achieving 2.74 GPa, and the yield strength increased by 86.7%, attaining 698 MPa. However, the ultimate tensile strength changed minimally, and the elongation rate decreased. These results are primarily due to the large number of dislocations in the sample.

(3) The high-pressure self-heating melting and quenching method is an exotic route to process a small piece of steel with moderate properties and ultrafine microstructure.

## Figures and Tables

**Table 1:** Chemical composition of the raw materials (wt %).

C	Cr	Ni	Si	Mn	Fe
0.06	18.12	8.72	0.41	1.42	balance

**Table 2:** Vickers hardness test results.

Sample	Hardness (GPa)	Standard Deviation
As-received SS304	2.06	0.09
Melted zone of PQ SS304	2.74	0.14
Non-melted zone of PQ SS304	1.94	0.11

**Table 3:** Tensile test results.

Sample	Yield strength (MPa)	Ultimate tensile strength (MPa)	Elongation (%)
As received SS304	374	780	44
PQ SS304	698	783	18
